# Protective effects of *Aureobasidium pullulans* lysate on UV-damaged human skin fibroblasts and HaCaT cells

**DOI:** 10.1186/s40643-023-00678-9

**Published:** 2023-08-28

**Authors:** Xin Wang, Yongtao Zhang, Dongdong Wang, Ning Su, Li Yang, Hao Fu, Jiachan Zhang, Meng Li, Changtao Wang

**Affiliations:** 1 Beijing Key Laboratory of Plant Resource Research and Development, College of Chemistry and Materials Engineering, Beijing Technology and Business University, Beijing, People’s Republic of China; 2 Institute of Cosmetic Regulatory Science, Beijing Technology and Business University, Beijing, People’s Republic of China; 3 Chinese Academy of Inspection and Quarantine, Beijing, People’s Republic of China; 4 Beijing Sino-German Union Cosmetic Institute Co., Ltd, Beijing, People’s Republic of China

**Keywords:** *A. pullulans* lysate, UV, Human skin fibroblasts, Human immortalized keratinocytes

## Abstract

**Background:**

*Aureobasidium pullulans* (*A. pullulans*) has a wide range of applications. Ultraviolet (UV) rays from the sun can cause skin photoaging. In order to explore the protective effect and application potential of *A. pullulans* lysate on UV-damaged human skin fibroblasts (HSF) and HaCaT Cells, this study investigates the anti-aging and anti-inflammatory effects of *A. pullulans* lysate as well as the mechanism of anti-oxidative stress at the cellular and molecular levels through cytotoxicity experiments, enzyme-linked immunosorbent assays (ELISA), and real-time quantitative PCR (RT-qPCR).

**Results:**

The experimental results have shown that the *A. pullulans* lysate can effectively reduce the loss of extracellular matrix components (EMC), such as collagen and hyaluronic acid (HA). It is also capable of scavenging excess reactive oxygen species (ROS) from the body, thereby increasing the activity of catalase, decreasing the overexpression of intracellular matrix metalloproteinases (MMPs), enhancing the gene expression of metalloproteinase inhibitors (TIMPs), and decreasing the level of inflammatory factors, reducing UV-induced apoptosis of HaCaT cells. Meanwhile, oxidative stress homeostasis is also regulated through the Nrf2/Keap1 and MAPK signaling pathways.

**Conclusions:**

This study shows that the *A. pullulans* lysate has the potential to resist photoaging.

**Graphical Abstract:**

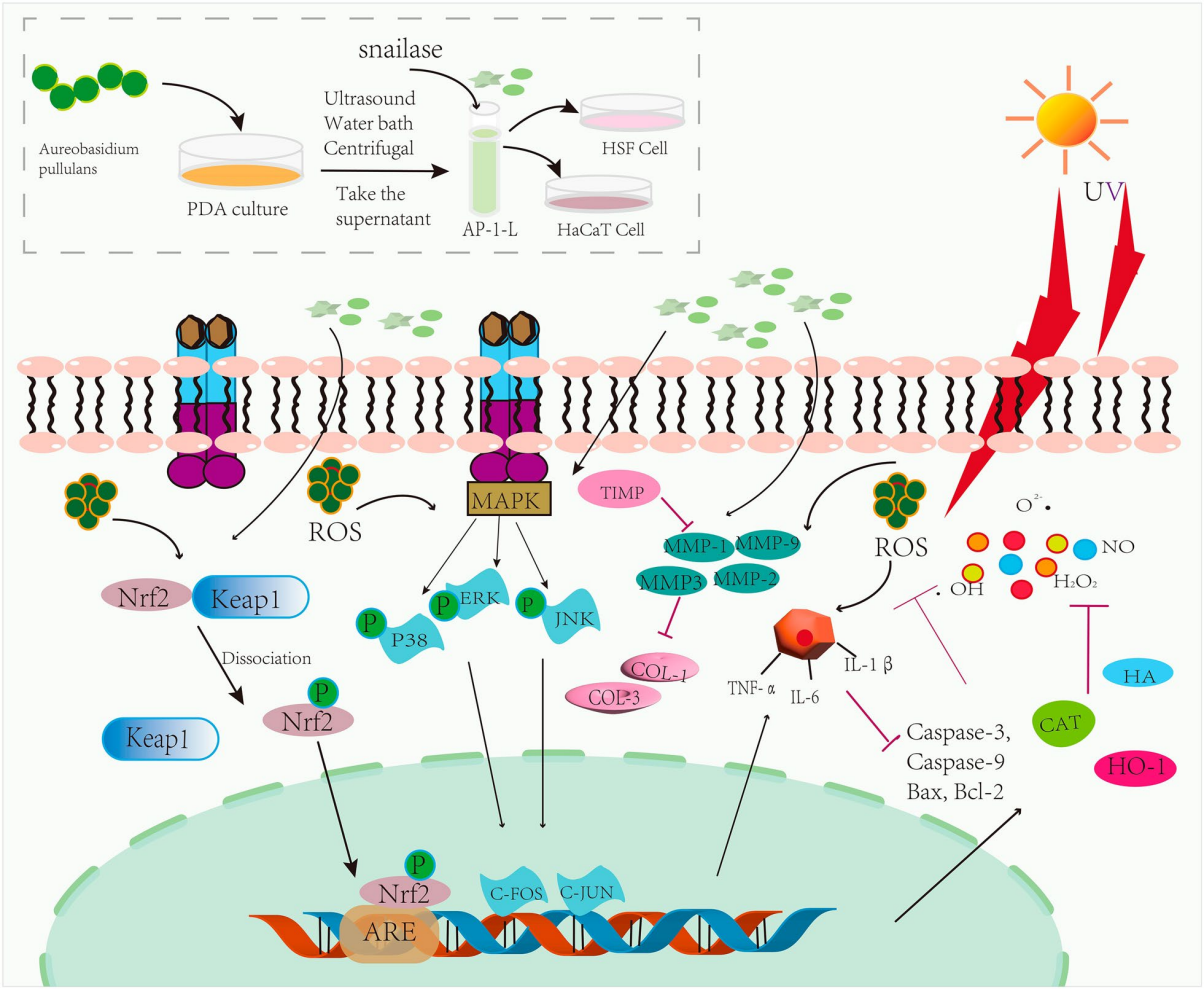

## Introduction

The skin, located at the most superficial layer of the organism, is a major barrier to pathogens and regulates moisture. Skin aging is affected by a number of factors, of which exogenous aging is mainly caused by ultraviolet (UV) radiation (Cavinato and Jansen-Durr 2017). UV rays from the sun include ultraviolet radiation a (UVA), ultraviolet radiation b (UVB), and ultraviolet radiation c (UVC), with wavelengths of 320–400 nm, 290–320 nm, and 100–290 nm, respectively. The UV rays that reach the earth's surface are composed of UVA and UVB. Among them, UVB reaches the earth's surface in a relatively low amount (about 5% of sunlight's UV rays). In contrast to UVB, UVA reaches the earth's surface in a larger proportion (about 95% of sunlight’s UV rays) (Fitsiou et al. 2021). These wave bands of UV radiation can penetrate the epidermis and even the dermis and interact with skin cells, affecting extracellular matrix (ECM) components and thereby causing cell metabolism changes, DNA damage, abnormal autophagy, enzyme activity changes, and the induction of oxidative stress. Of these, the most serious damage to the skin structure reached 90%, including photoaging, photo-immunosuppression, photo-carcinogenesis (Rysava et al. 2020). UVA has the strongest penetrating power, enabling it to reach the dermis of human skin, where it can accelerate wrinkles, pigmentation, dryness, and decreased elasticity, promote the increase of ROS and matrix metalloproteinases (MMPs) in dermal cells (Lepetsos and Papavassiliou 2016), and cause the degradation of the ECM. UVB is the most damaging to the skin. Long-term UVB irradiation can cause skin relaxation, roughness, and erythema. UVB-induced skin damage is primarily associated with inflammation, tumor necrosis factor content, and ECM degradation due to the release of proinflammatory cytokines. It also disrupts the skin barrier by causing a molecular chain reaction that up-regulates the expression of MMPs in the dermis and epidermis and degrades ECM components. Therefore, preventing the degradation of the extracellular matrix of the skin, alleviating the inflammatory response, and resisting oxidative stress are effective ways to inhibit UV-induced skin photoaging.

Research on the use of postbiotics in the skin has emerged in recent years. Postbiotics are soluble factors (products or metabolic by-products) secreted by live bacteria or released by bacterial lysis, including short-chain fatty acids, peptidoglycans, functional enzymes, peptides, extracellular/wall polysaccharides, organic acids, and bacteriocins. Notably, the microbial community specified by postbiotics is not limited to probiotic species but also includes beneficial components contained in or secreted by other bacterial species (Aguilar-Toala et al. 2018). More importantly, postbiotics have better safety and stability, better absorption, and metabolic advantages (Cuevas-Gonzalez et al. 2020). And there is data showing that it has antibacterial, antioxidant, and immunomodulatory effects (Sharma and Shukla 2016). Among them, microbial lysate products and metabolites have multiple uses and have been applied in the field of cosmetics. Studies have shown that *Bifida* ferment lysate can repair the damage to the muscle bottom, protect against UV damage, and prevent photoaging. *Lactic acid bacteria’s* fermentation cytolytic products have antibacterial, moisturizing, reparative, and anti-inflammatory effects. The extracellular metabolites produced by *Saccharomyces boulardii* have rich biological activities, showing high antioxidant, antibacterial, and anti-inflammatory properties (Fu et al. 2023a). *Lactobacillus reuteri* SJ-47 strain exopolysaccharides can improve antioxidant capacity and delay skin photoaging caused by UVA radiation (Zhao et al. 2022). It can be seen from the above that probiotics have a wide range of applications. The next few years will see new vigor in the research and applications of this field.

*Aureobasidium pullulans* (*Aureobasidium *spp.), commonly known as black yeast (Zou et al. 2019), is a kind of dark yeast-like fungus prevalent in nature (Panzer et al. 2021), which can produce melanin (Wei et al. 2021), pullulan polysaccharides (Liu et al. 2021), -glucan, -polymalic acid (Wei et al. 2019), enzymes β-d-fructofuranosidase (Khatun et al. 2021), heavy oil, ferrophilin, massoia lactone, lactic acid, etc. This indicates that *A. pullulans* has an abundant active product that merits further investigation. However, domestic and international research on *A. pullulans* has focused on the development of food and pharmaceutical applications for the fermented product, and less research has been done on its lysates. Recently, there have been studies on the skin regenerating and soothing effects of *A. pullulans*, extracts of its lysates, or culture products, which illustrate the value and significance of further development and application of *A. pullulans* (Hohyun and Kilsun 2022). But there have been no experimental studies of the protective effects of *A. pullulans* lysate on UV-damaged human skin fibroblasts and HaCaT cells. Based on the promising prospect of postbiotics in the future cosmetic industry as well as the wide range of sources and abundant metabolites of *A. pullulans*, as well as the necessity of defence against photoaging, this study will investigate whether *A. pullulans* lysate have antioxidant and anti-inflammatory effects, and whether *A. pullulans* lysate have a protective effect on two types of cells under UV radiation.

In this study, *A. pullulans* lysate was first obtained by culture and preparation. Subsequently, the anti-aging and anti-inflammatory effects of *A. pullulans* lysate have been investigated by assessing cytotoxicity, changes in ROS, and by studying the content and relative expression of oxidative and inflammatory factors in cells by ELISA and RT-qPCR. Next, its efficacy was further validated by Nrf2/Keap1 signaling pathway and MAPK signaling pathway. The aim of this paper is to explore the protective effects of *A. pullulans* lysates on UV-damaged HSF and HaCaT cells, as well as their potential applications and development prospects in cosmetics.

## Materials and methods

### Materials

*Aureobasidium pullulans* (China General Microbiological Culture Collection Center, CGMCC 3.3984); human skin fibroblasts (HSFs) were provided by Shanghai Institute of Biological Sciences; human immortalized keratinocytes (HaCaT) were provided by Cell Resource Center of Peking Union Medical College.

PDA medium (Qingdao Hope Bio-Technology Co., Ltd.); Fetal bovine serum (FBS); Dulbecco’s modified Eagle’s medium (DMEM); trypsin–EDTA; penicillin–streptomycin; phosphate buffered saline (PBS, Solarbio Technology, Beijing, China); Cell Counting Kit-8 (CCK-8) (China Liji Biotechnology Co., Ltd.); UV light box (Spectronics Co., Ltd., USA); catalase (CAT) ELISA Kit (China Biyuntian Company); BCA Protein Quantitative Assay Kit, Type I Collagen (COL-I) ELISA Kit, Hyaluronic Acid (HA) ELISA Kit (Nanjing Jiancheng Biology, China); (TNF-α, IL-6, IL-1β) ELISA kits (Nanjing Jiancheng Bioengineering Institute, China); (Caspase-3 activity assay ELISA kit, Caspase-9 activity assay ELISA kit, BCL-2 related X protein ELISA kit, B cell lymphoma factor-2 ELISA kit) (Beijing Bailuigi Biotechnology Co., LTD); Human Matrix Metalloproteinase-1 (MMP-1) ELISA kit, Human Matrix Metalloproteinase-1 (MMP-2) ELISA Kit, Human Matrix Metalloproteinase-1 (MMP-3) ELISA kit, Human Matrix Metalloproteinase-1 (MMP-9) ELISA kit and metalloproteinases inhibitor (TIMP) ELISA kit (CLOUD-CLONE CORP, USA); qPCR-related kit (Baiyuan Biotechnology (Beijing) Co., Ltd.).

### Preparation of lysis products of *A. pullulans*

Firstly, *A. pullulans* (provided by the China General Microbiological Culture Collection Center CGMCC) was activated by PDA slant medium (potato 200 g/L, glucose 20 g/L, agar 20 g/L, pH 5.4–5.8) in a constant temperature incubator at 28 ℃. Then, 1 ring spore was selected and inoculated into the liquid medium, and the cell suspension was cultured at 34 ℃, 180 rpm/min, until the absorbance of the cell suspension at 600 nm reached 1–1.2. Get a solution of bacteria.

The above 50 mL of bacterial solution was centrifuged (10,000 rpm, 10 min) to obtain the cell precipitate, which was completely mixed with 20 mL of distilled water. Ultrasound destruction of cells was performed in an ice bath (operating frequency of 25%, ultrasound for 15 s, interval of 20 s, total duration of 20 min) using a cell ultrasonic crusher (Jiangsu Wave Field Intelligent Technology Co., Ltd.). Subsequently, snailase was added to destroy the cell wall, followed by heating in a water bath (80 ℃ 30 min) to inactivate the snailase, and then cooling and centrifugation (10,000 rpm, 10 min). The supernatant was collected to obtain the lysate, named “AP-1-L”, and stored in the refrigerator at – 20 ℃.

### Cell culture

The HSF used in the whole experiments were 5–10 generations, and the HaCaT used were 5–20 generations. HSF cells and HaCaT cells were cultured in DMEM medium (containing 10% FBS and 1% penicillin–streptomycin), respectively. The cultural environment was a constant temperature incubator (BPN-50CH(UV), Shanghai Yiheng Scientific Instrument (Shanghai, China) Co., Ltd.) with 37 ℃ and 5% CO^2^. The cells were cultured for 2–3 days and subcultured. Follow-up experiments can be carried out when the cell fusion rate is greater than 80%.

### Cell viability

The effect of AP-1-L on the viability of HSF cells and HaCaT cells. The toxicity levels of AP-1-L in the two types of cells were detected using the CCK8 method. According to the instructions of the CCK8 kit, the cell suspensions (HSF cells, HaCaT cells) were inoculated with 1 × 10^4^ cells/well in a 96-well plate. When the cell density in the 96-well plate was 70–80%, cells were treated with different concentrations of samples (the concentration gradient was 0.3125%, 0.625%, 1.25%, 2.5%, 5%, 10%, 20%, 4%), each sample was made six-parallel, and the culture continued for 24 h. Among them, the blank group was incubated with serum-free DMEM for 24 h. After 24 h of incubation, 100 μL serum-free DMEM was added to each well after washing with PBS, and 10 μL CCK8 was cultured at 37 ℃ for 2 h. The absorbance was then measured at the 450 nm wavelength. In addition, when AP-1-L had a volume fraction of 1%, the cell survival rate was over 80% and the cytotoxicity was low. Therefore, this volume fraction was used in the subsequent experiments.

UVA dose screening. The well-grown HSF cells were inoculated in cell culture plates at a concentration of 8 × 10^3^ cells/well. The cells were cultured in the cell incubator for 12 h. Then the original special medium for HSF cells was discarded and the original special medium for HSF cells was replaced with serum-free DMEM medium. The DMEM medium was replaced with appropriate PBS before UVA irradiation. Then the UVA light box (Spectronics Co., Ltd., USA) was used to irradiate the cells with UVA doses of 1, 2, 3, 4, 5, 6, 7, 8, 9, and 10 J/cm^2^, respectively. When the UVA dose was 7 J cm^2^, the cell survival rate was about 80%. We chose 7 J/cm^2^ as the UVA damage model.

UVB dose screening. The well-grown HaCaT cells were inoculated in cell culture plates at a concentration of 8 × 10^3^ cells/well. The cells were cultured in the cell incubator for 12 h. Then the original special medium for HaCaT cells was discarded, and the original special medium for HaCaT cells was replaced with serum-free DMEM medium. The DMEM medium was replaced with appropriate PBS before UVB irradiation. Then the UVB light box (Spectronics Co., Ltd., USA) was used to irradiate the cells with UVB doses of 10, 20, 30, and 40 mJ/cm^2^, respectively. When the UVB dose was 16 mJ/cm^2^, the cell survival rate was about 80%. We chose 16 mJ/cm^2^ as the UVB damage model.

### Determination of ROS in HSF cells

HSF cell suspension (2 × 10^5^ cells/well) was added to each well of the 6-well plate, followed by 2 mL DMEM for dilution. AP-1-L and VC were added to the sample group and the positive control groups, respectively. All but the blank control groups were irradiated with UVA. The cells were cultured in an incubator for 12 h, stimulated with 7 J/cm^2^ UVA, incubated for 12 h and gently washed with PBS, then given a fresh medium of 1 mL DCFH-DA (DCFH-DA:DMEM = 1:1,000 serum-free medium dilution). After incubation at 37 ℃ for 20–30 min, the cells were gently washed in PBS 1–2 times to remove excess the DCFH-DA. Finally, they were observed and photographed under a confocal microscope (CKX53, Olympus LS, Tokyo, Japan).

### Enzyme-linked immunosorbent assay (ELISA)

ELISA is an immunoenzyme technique developed after immunofluorescence and radioimmunoassay. It has high sensitivity, good reproducibility and strong stability. The content of related factors in cells was determined by ELISA.

### Determination of BCA, CAT, HA and COL-I in HSF cells

Preparation of cell lysate supernatant: as in the above cell culture steps, the cells in a good growth state were counted and inoculated in 6-well plates with 2 × 10^5^ cells per well. The cells were cultured overnight at 37 ℃ and 5% CO_2_, then the medium was discarded. 2 mL AP-1-L and 2 mL VC solutions were added to the sample group and the positive control group, respectively. The blank control group and the model group were added to the same volume of serum-free DMEM and cultured for 24 h. Then the model group, the sample group and the positive control group were given UVA radiation. After irradiation, the PBS was washed twice and then discarded. After 12 h of culture with serum-free DMEM, PBS was washed twice, and then 200 μL of lysis solution was added to each well. When the cells are fully lysed, they are scraped by a scraper and collected into a centrifuge tube. The cells were centrifuged at 4 ℃ for 2 min to obtain the cell lysis supernatant. The collected cell lysate was used to determine the protein content of each group using the BCA protein content detection kit.

According to the instructions of the kits, the enzyme activity of catalase (CAT), COL-I protein, and HA in HSF cells were detected using the ELISA kit. The contents of the supernatant were detected using the CAT ELISA kit (Nanjing Jiancheng Biology, China), the COL-I protein ELISA kit (Nanjing Jiancheng Biology, China) and the HA ELISA kit (Nanjing Jiancheng Biology, China) using the cell lysate supernatant collected as described above.

### Determination of inflammatory factors and apoptosis factors in HaCaT cells

Inflammatory factors and apoptotic factors in HaCaT cells were detected using ELISA kits according to the kit instructions. Through the above-collected cell lysate supernatant, IL-1β ELISA kit, IL-6 ELISA kit, TNF-α ELISA kit, Caspase-3 ELISA kit, Caspase-9 ELISA kit, Bax ELISA kit, Bcl-2 ELISA kit were used to detect the content of the supernatant.

### Real-time fluorescence quantitative PCR

The well-grown HSF cells were inoculated into a 6-well culture plate at a density of 2 × 10^5^ cells/well and cultured for 12 h. Subsequently, the medium was discarded, washed with PBS, and then 2 mL of AP-1-L (1% *v*/*v*) was added. Meanwhile, the same volume of serum-free DMEM was added to the blank control group and the model group. Similarly, the same volume of VC was added to the positive control group. The cells were incubated for 24 h. At the end of the incubation, all cells except the blank control group were given UVA radiation, followed by the addition of 2 mL of serum-free DMEM. 12 h later, the cells were washed with PBS and collected for subsequent experiments. Total RNA was extracted using Trizol, and the gene expression of MMP1, MMP2, MMP3, MMP9, TIMP1, COL-I, COL-III, and related factors in Nrf2/Keap and MAPK signaling pathways were detected by RT-qPCR in HSF cells.

The well-grown HaCaT cells were inoculated into a 6-well culture plate at a density of 2 × 10^5^ cells/well and cultured for 12 h. Subsequently, the medium was discarded, washed with PBS, and then 2 mL of AP-1-L (1% *v*/*v*) was added. Meanwhile, the same volume of serum-free DMEM was added to the blank control group and the model group. Similarly, the same volume of VC was added to the positive control group. The cells were incubated for 24 h. At the end of the incubation, all cells except the blank control group were given UVB radiation, followed by the addition of 2 mL of serum-free DMEM. 12 h later, the cells were washed with PBS and collected for subsequent experiments. Total RNA was extracted using Trizol, and the gene expression of inflammatory factors TNF-α, IL-6, IL-1β, and apoptotic factors Caspase3, Caspase9, Bax, and Bcl-2 in HaCaT cells was detected using RT-qPCR.

### Statistical analysis

The experimental data were analyzed by GraphPad Prism8 data software. All numerical data were expressed as mean ± standard deviation, and the *P* value indicated the difference between groups.

## Results

### Effects of AP-1-L on activity of HSF and HaCaT cells

**Fig. 1:**
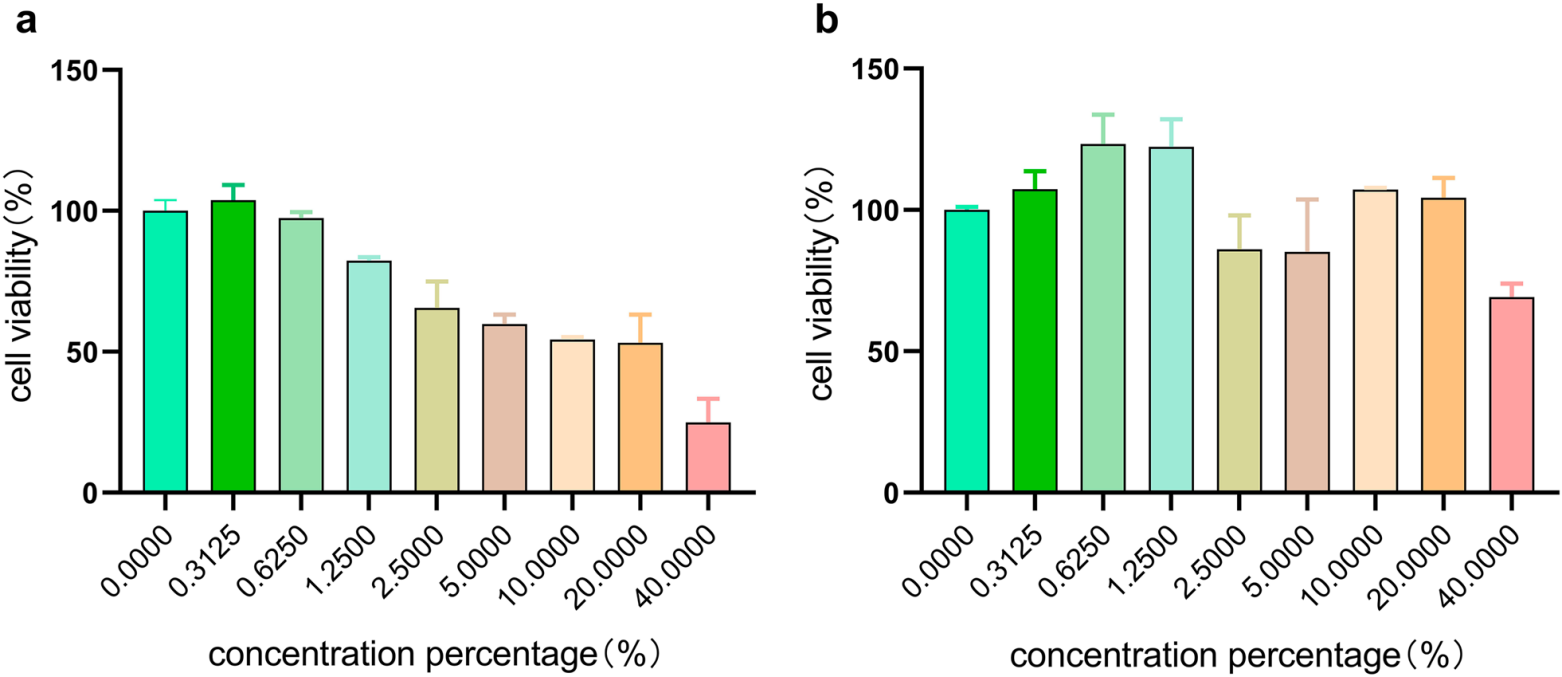
Effects of AP-1-L on HSF and HaCaT cell viability: **a** effects of different concentrations of AP-1-L on HSF cell viability; **b** effects of different concentrations of AP-1-L on HaCaT cell viability

The cytotoxicity levels of AP-1-L on HSF and HaCaT cells were measured by CCK8, as well as selecting the right concentration for the sample **(**Fig. 1a and b). Figure 1a shows that AP-1-L had almost no toxic effect on HSF cells cultured with a volume fraction of less than 1.25%, and the survival rate of HSF cells reached more than 100% at a volume fraction of 0.3125%. Figure 1b shows that AP-1-L had almost no toxic effect on HaCaT cells, and the cell viability of HaCaT cells cultured in AP-1-L with a volume fraction of 1.25% was more than 100%. It was concluded that the survival rates of the two types of cells cultured in AP-1-L before the volume fraction of 1.25% were high, and there was almost no toxic effect. Finally, considering the effect of AP-1-L on the viability of HSF and HaCaT cells, AP-1-L with a volume fraction of 1% was selected as the subsequent experimental concentration.

### Inhibitory effects of AP-1-L on ROS production

**Fig. 2:**
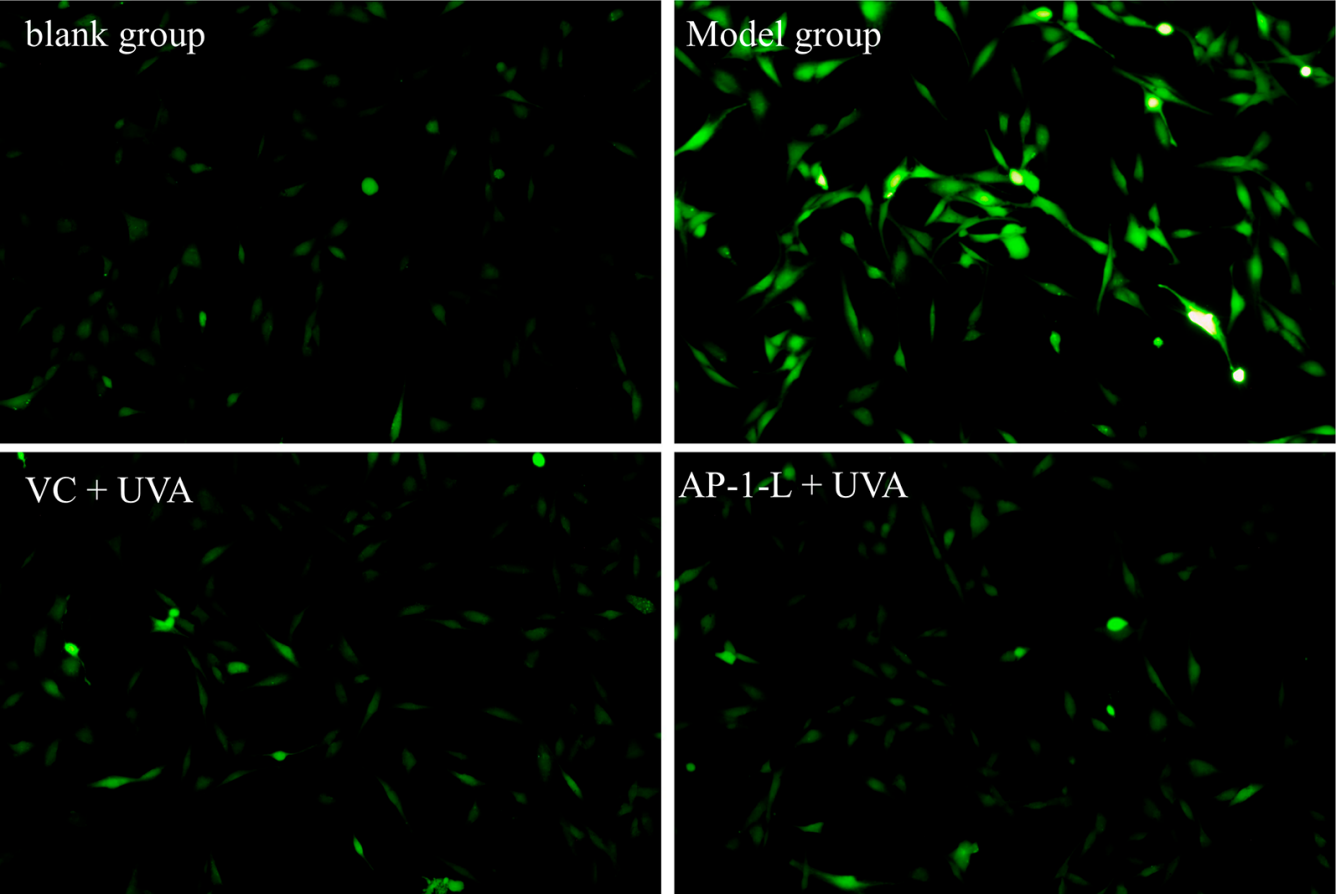
The fluorescence photo of intracellular ROS

UV radiation can stimulate the production of ROS, which is involved in cell growth, development, senescence and apoptosis. It plays a crucial role in mediating various biological reactions and can activate multiple signaling pathways **(**Schuch et al. 2017). In our studies, ROS content in cells was detected by the fluorescent probe DCFH-DA. The higher the ROS content in the cell, the stronger the fluorescence intensity, and thus the antioxidant activity of the sample is further explained by looking at the fluorescence intensity of the sample before and after the treatment. As shown in Fig. 2, the blank group demonstrates the amount of ROS normally contained in the cells, and it is obvious from the picture that there is almost no fluorescence, indicating a low level of ROS. In the model group, fluorescence intensity was significantly enhanced after UVA irradiation, indicating that UV radiation can induce intracellular ROS generation. The fluorescence intensity of the cells in the sample group and the positive control group was significantly reduced compared with the model group, indicating that the addition of the sample followed by UV irradiation could reduce the production of ROS, further suggesting that AP-1-L has a protective effect on UV-damaged HSF cells. Compared with the sample group and the blank group, the fluorescence intensity was basically the same, indicating that the addition of AP-1-L could result in the recovery of intracellular ROS to the original level. It was concluded that AP-1-L efficiently scavenges and inhibits intracellular excess ROS and maintains oxidative stress balance to protect cells from damage.

### Effects of AP-1-L on ECM components (CAT, HA and COL-I)

UV radiation can damage the composition of the skin's extracellular matrix (Franco et al. 2022). Skin aging and damage are related to moisture and protein loss. HA as one of the components of ECM, which is of great significance for maintaining skin water content and can be used as a criterion for judging the degree of skin aging. CAT is an antioxidant enzyme that is very important in maintaining balance between the oxidation and peroxide systems. COL-I maintains cellular elasticity.

**Fig. 3:**
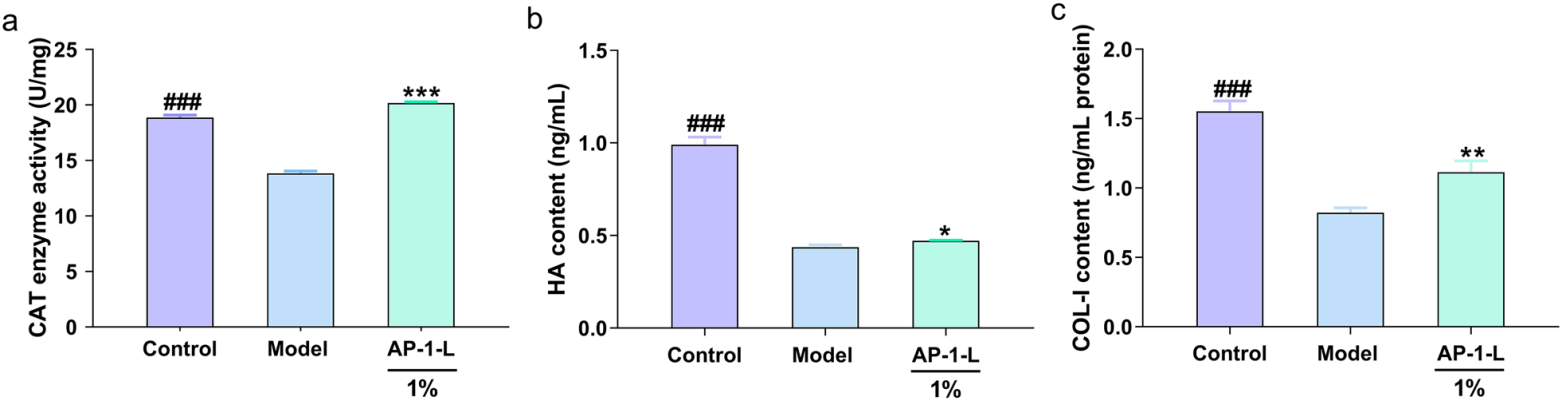
Effects of AP-1-L on CAT, HA and COL-I: **a** CAT enzyme activity; **b** HA content; **c** COL-I protein content. Significant difference analysis: comparison between each group and model group. (*P < 0.05, **P < 0.01, ***P < 0.001, difference analysis between sample group and UVA model group; ^##^*P* < 0.01, ^###^*P* < 0.001, difference analysis between blank control group and UVA model group.)

In this study, the contents of CAT, HA and COL-I in cells were detected by ELISA. As shown in Fig. 3a, after 7 J/cm^2^ UVA radiation, the CAT content in the cells was significantly lower than that in the blank group (*P* < 0.001), indicated that CAT activity was inhibited by UVA radiation. When AP-1-L was added and then UVA radiation was applied, the CAT content in the cells was significantly higher than that in the model group (*P* < 0.001). It can be seen that AP-1-L has better antioxidant activity. As shown in Fig. 3b, the HA content in HSF cells decreased significantly after UVA irradiation, while it increased slightly in cells treated with AP-1-L. From Fig. 3c, it can be seen that the content of COL-I was significantly reduced when UVA radiation was applied, indicating that the loss of COL-I in the skin was caused by UVA radiation and that the content of COL-I was restored by adding AP-1-L.It has been shown that UVA significantly reduces the ECM content, and AP-1-L is able to reduce the ECM content loss after UV irradiation. It was concluded that AP-1-L increases the CAT and HA content in HSF cells and protects organelles from oxidative damage.

### Effect of AP-1-L on inflammatory and apoptotic factors in HaCaT cells

**Fig. 4:**
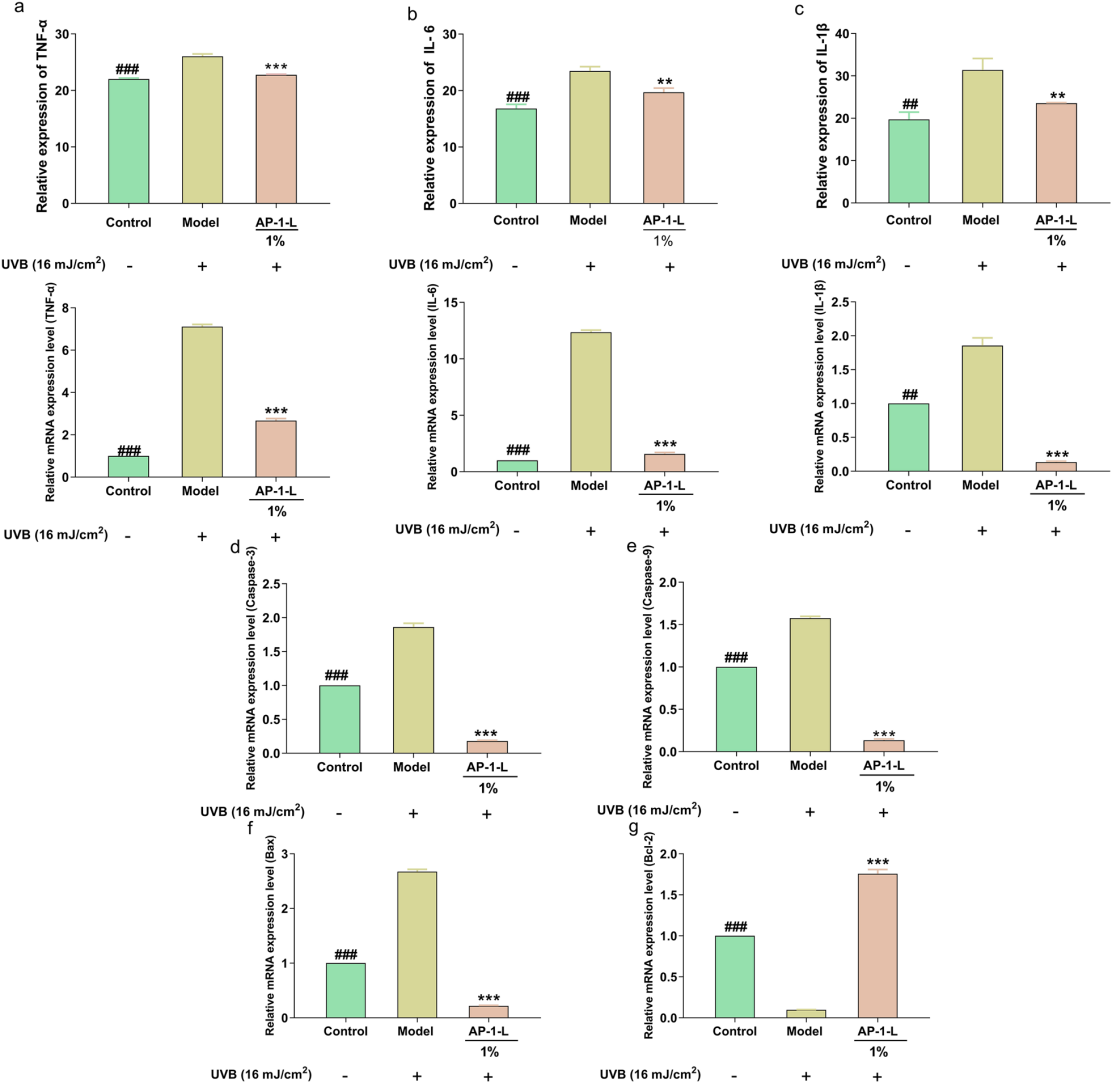
Effect of AP-1-L on inflammatory and apoptotic factors in HaCaT cells: **a**–**c** effects of AP-1-L on TNF-α, IL-8 and IL-1β and mRNA relative expression levels. **d**–**g** Effects of AP-1-L on Caspase-3, Caspase-9, Bax, Bcl-2. Significant difference analysis: comparison between each group and model group. (**P < 0.01, ***P < 0.001, difference analysis between sample group and UVA model group; ^##^*P* < 0.01, ^###^*P* < 0.001, difference analysis between blank control group and UVA model group.)

Exposure to UVB radiation can cause DNA damage in HaCaT cells and release signals that activate and produce various inflammatory factors such as cytokines IL-1α, IL-6 and TNF-α. As shown in Fig. 4a–c, it can be seen that the contents of inflammatory factors IL-1β, IL-6 and TNF-α increased in the cells after UVB irradiation, and decreased in cells treated with AP-1-L. The results indicated that UVB produced inflammatory damage to HaCaT cells; whereas AP-1-L could reduce the content of inflammatory factors or even tend to normal levels. Further, RT-qPCR was used to detect the expression changes of three inflammatory factors at the transcriptional level. When UVB irradiated HaCaT cells, the expressions of three inflammatory genes were up-regulated. When AP-1-L was added and then UVB radiation was applied, the expressions of the three genes were significantly down-regulated. The occurrence of apoptosis is closely related to inflammation. The expression levels of four apoptosis factors were detected by RT-qPCR. As shown in Fig. 4d–g, after HaCaT cells were exposed to UVB radiation, the apoptotic factor (Caspase-3, Caspase-9, Bax) gene expression was increased and Bcl-2 gene expression was decreased compared with the blank group. When AP-1-L was added, the expressions of Caspase-3, Caspase-9 and Bax genes were significantly decreased, and the expression of Bcl-2 gene was up-regulated. The results showed that AP-1-L could down-regulate the expression of apoptosis factor gene. It was concluded that AP-1-L effectively modulates the UVB-induced inflammatory and apoptosis response of HaCaT cells, exhibiting anti-inflammatory effects and effectively mitigating cell damage.

### Effects of AP-1-L on collagen and matrix metalloproteinases

**Fig. 5:**
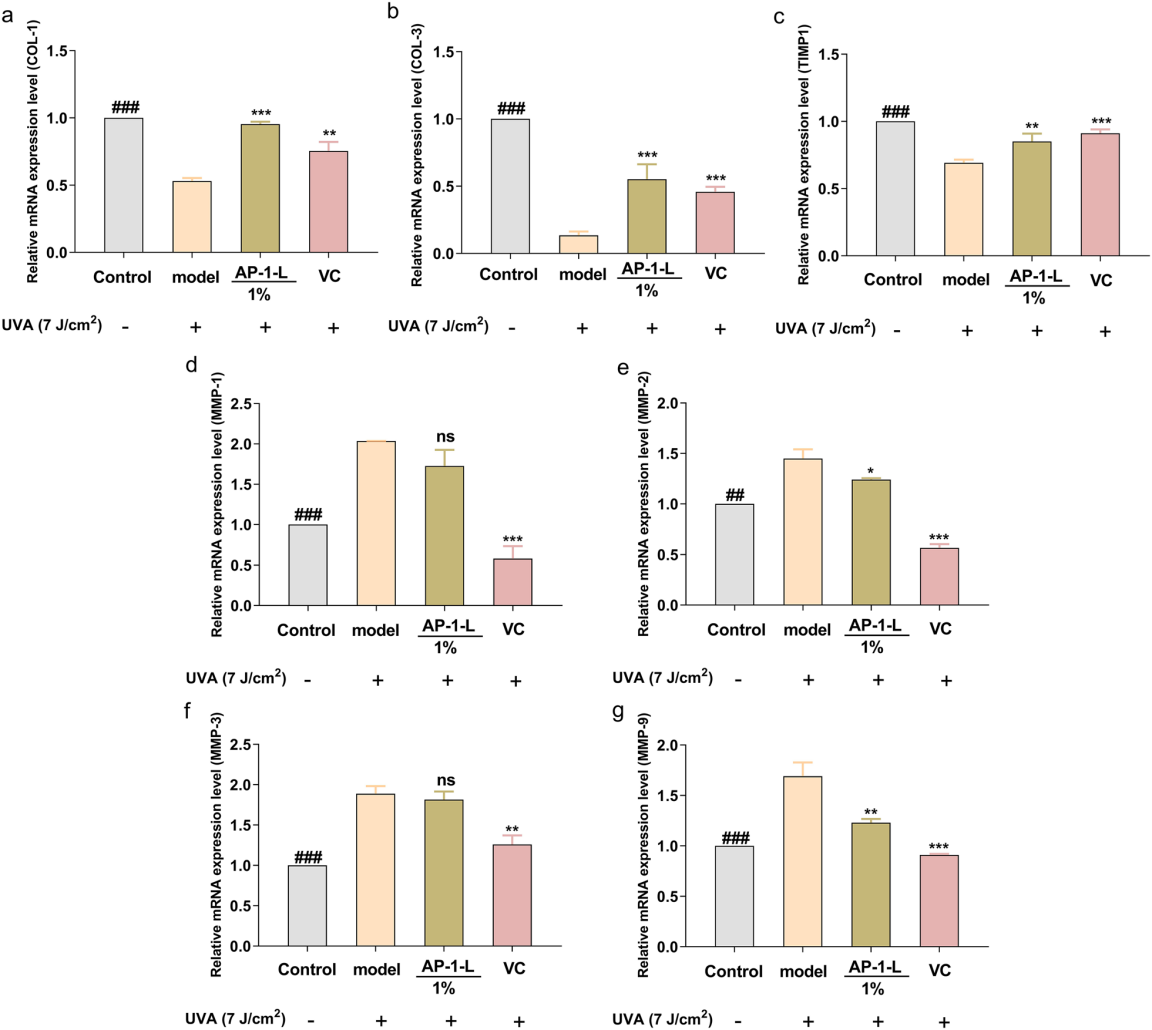
Effects of AP-1-L on collagen and matrix metalloproteinases: **a**, **b** relative expression of Type I and III collagen mRNA; **c**–**g** relative expression of TIMP1, MMP-1, MMP-2, MMP-3 and MMP-9 mRNA. (*P < 0.01, **P < 0.05, ***P < 0.001, difference analysis between sample group and UVA model group; ^##^*P* < 0.05, ^###^*P* < 0.001, difference analysis between blank control group and UVA model group.)

MMPs are among the main factors involved in photoaging-related skin changes. As shown in Fig. 5a–c, the content and gene expression of COL-I and COL-III in AP-1-L-treated cells were significantly reduced after UVA irradiation, suggesting that UVA irradiation induced skin damage and loss of collagen, whereas the content and expression of collagen were enhanced in AP-1-L-treated cells, which was stronger than that in the positive control group, suggesting that AP-1-L was able to resist cellular damage brought about by UVA radiation damage caused by UVA radiation. Figure 5d shows that the expression of TIMP decreased after UVA irradiation and was up-regulated by AP-1-L treatment. It can be seen from Fig. 5e–h that the expression of MMPs in the cells treated with AP-1-L decreased. MMPs activity is inhibited by endogenous TIMP, which is an important regulator of MMPs synthesis and degradation. Normally, TIMPs bind to MMPs to form complexes that regulate the activation of MMPs. When exposed to UV radiation, the mRNA expression level of intracellular MMPs was significantly increased, resulting in the decrease of TIMP-1, whereas AP-1-L treatment inhibited the overexpression of MMPs, thus restoring the expression of MMPs and TIMP to the original level. In addition, the effect of VC was more pronounced compared to the positive control group. VC, as a recognized antioxidant substance, has strong antioxidant activity, and this experiment shows that VC was more effective than AP-1-L in regulating the balance between MMPs and TIMP1 activities. However, it can also be shown that AP-1-L works well. It was concluded that AP-1-L enhances protein content and related gene expression levels in HSF cells while modulating expression levels of TIMP and MMPs.

### Effects of AP-1-L on mRNA expression

Nrf2 is a transcription factor that plays a key role in the oxidative stress response. The enhanced expression of the Nrf2 gene can effectively delay and alleviate oxidative stress. ERK, p38, and JNK are three branches of the MAPK signaling pathway, which is involved in many processes of cell activity.

**Fig. 6:**
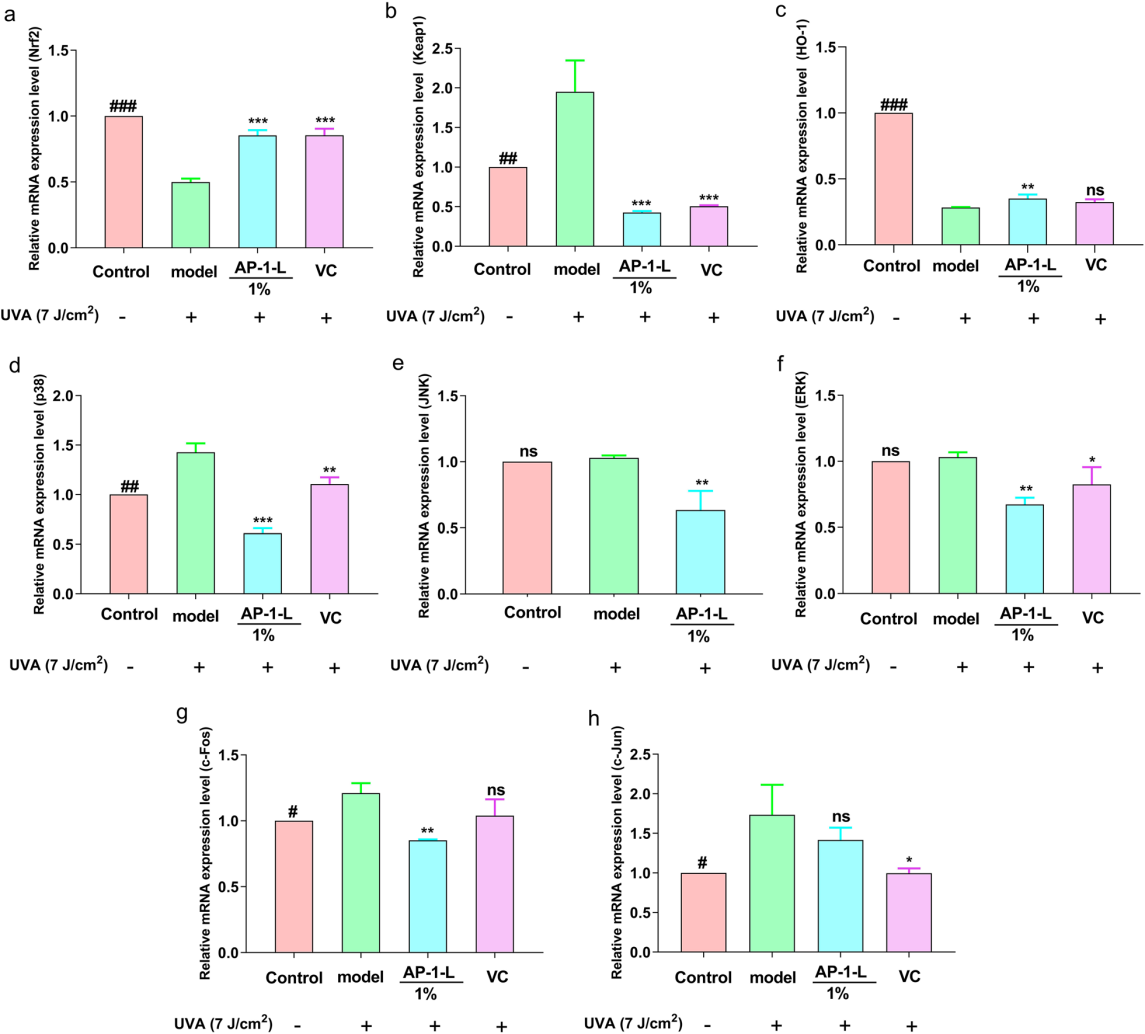
Effects of AP-1-L on Nrf2/Keap1 pathway and MAPK pathway gene expression in HSF: **a**–**h** effects of AP-1-L at 1% concentration on Nrf2, Keap1, HO-1, JNK, ERK, p38, c-Fos and c-Jun. (*P < 0.01, **P < 0.05, ***P < 0.001, difference analysis between sample group and UVA model group; ^#^*P* < 0.05, ^##^*P* < 0.01, ^###^*P* < 0.001, difference analysis between blank control group and UVA model group.)

As shown in Fig. 6a–c, UVA irradiation can significantly down-regulate the expression of the Nrf2 gene and the HO-1 gene and up-regulate the expression of the Keap1 gene. When AP-1-L was added and then UVA radiation was applied, the expression of the Nrf2 gene and the HO-1 gene were up-regulated, and the expression of the Keap gene was down-regulated. As shown in Fig. 6d–h, compared with the blank group, UVA irradiation significantly enhanced the expression of the JNK, ERK, p38, c-Fos, and c-Jun genes, which were significantly decreased after AP-1-L treatment, and the effect was stronger than the positive control. It is noteworthy that the positive control in Fig. 6e did not work, possibly because VC does not play a role in regulating JNK mRNA expression levels. Overall, AP-1-L has a better ability to regulate pathways and reduce cellular damage from UV radiation.

## Discussion

The UV component of sunlight can cause various damages to human skin. Excessive exposure to UV radiation will increase the formation of ROS and destroy the redox balance in skin cells. In addition, higher concentrations of ROS will damage the main protein components that constitute the skin, such as collagen (Perez-Sanchez et al. 2014; Rinnerthaler et al. 2015), regulate the synthesis and metabolism of the ECM, induce inflammation, mediate cell signaling pathways, inhibit cell proliferation, induced apoptosis and even lead to cell death (Atalay et al. 2020). The exopolysaccharides, heavy oils, and polymalic acid produced by *A. pullulans* have been applied in the field of cosmetics (Zou et al. 2021; Zou et al. 2019). However, AP-1-L, which has not yet been reported in the field of cosmetics, possesses intracellular extracts with biosimilar structures, such as cytoplasm, cell wall, and polysaccharide complexes. Such extracts contain beneficial skin care molecules required by biological cells, such as vitamins, minerals, and amino acids of trophoblasts, and can penetrate the skin barrier, repair damage to the bottom of the muscle, and prevent damage caused by UV light, thereby preventing aging. Just as the metabolites of multiple classes of bacteria have been extensively studied, the soluble fraction of bacterial cells has received little attention and few of their findings have been reported, but scientific evidence for their health benefits (e.g., providing physiological benefits to the host) is gradually increasing (Compare et al. 2017). Therefore, AP-1-L has good research value. In this study, the photoprotective effect of AP-1-L on HSF and HaCaT cells under UV irradiation was evaluated by detecting and analyzing the contents and expression levels of extracellular matrix components, inflammatory factors, and oxidative stress-related factors in cells, exploring the application potential of AP-1-L.

Under normal circumstances, ROS in the body maintains a dynamic balance. Nevertheless, when ROS content increases, the body's dynamic balance is broken, causing a range of hazards (Fu et al. 2023b). In this study, UV irradiation of HSF cells produced excess ROS, leading to oxidative stress and the increased expression of MMPs at the molecular level, which promoted the degradation of the ECM, reduced normal collagen content, and weakened CAT expression (Pyo et al. 2005). Studies have shown that CAT is an important antioxidant enzyme for preventing and alleviating peroxide stress reactions (Li et al. 2022). It can reduce ROS to H_2_O to remove excess ROS, and it is essential to maintain the balance between oxidation and peroxide systems (Zhang et al. 2019). HA is a kind of natural polysaccharide, and its content in the epidermis and dermis accounts for about 50% of the total HA content in the human body. It has a strong ability to bind water molecules and plays a role in removing ROS in cells, maintaining tissue homeostasis, and providing mechanical support. When cells are damaged, a large amount of HA is synthesized and degrades under the action of ROS (Arianna et al. 2018). Collagen can promote cell repair and maintain hydration, and the decrease in its content accelerates cell damage. In the ECM of the skin, type I collagen is a functional protein with the highest content, which is of great significance for maintaining skin elasticity, and its content can be used as a criterion for judging the degree of skin aging (Chen et al. 2019). TIMP and MMPs are important modulators of ECM balance. When cells are damaged by UV radiation, MMPs can promote the degradation of the ECM. Among them, MMP-3 will cooperate with MMP-1 and MMP-9 to degrade collagen, eventually leading to structural changes and functional loss of extracellular matrix components in the skin, causing the skin to lose elasticity, form wrinkles, and prematurely age (Xiao et al. 2020). However, in cells treated with AP-1-L, ROS content decreased significantly and CAT, HA, and COL-I contents increased compared with cells damaged by UV radiation. All of these results suggest that AP-1-L may play a protective role involving antioxidation, inhibiting ECM degradation, and reducing the UVA-induced production of MMPs.

Inflammatory factors can promote the production of ROS in cells, accelerate the activation of MMPs and the degradation of collagen, and promote skin aging. UVB radiation causes an increase in skin damage and inflammation due to an increase in the amount of inflammatory factors secreted by keratinocytes as inflammation occurs, which is one of the representative immune responses to inflammatory stimuli. TNF-α, IL-6, and IL-1β are key regulators of inflammation. Changes in the levels of three inflammatory factors measured at the protein level using ELISA. After UVB irradiation, the inflammatory factors in HaCaT cells were significantly elevated with respect to the blank population, indicating that UVB produced inflammatory damage in HaCaT cells. Thankfully, the levels of TNF-α, IL-6, and IL-1β were significantly reduced in UVB-irradiated cells after the addition of AP-1-L. The results indicate that AP-1-L can reduce cell damage by decreasing the secretion of proinflammatory factors. On the other hand, the occurrence of apoptosis is closely related to inflammation. Caspase-9 is the initiator of apoptosis and activates the effector Caspase-3, which then activates DNA enzyme to start the apoptosis process (Tsuchiya et al. 2020). Bcl-2 can play an anti-apoptotic role, and the overexpression of Bcl-2 can lead to changes of the REDOX balance in the nucleus, thereby reducing the activity of Caspase. Bax is a member of the Bcl-2 family involved in apoptosis, which can induce apoptosis, and the Bax/Bcl-2 ratio is a switch that initiates apoptosis (Akar et al. 2023; Eraslan et al. 2021; Li et al. 2019) The expression of cells and apoptosis factors (Caspase-3, Caspase-9, Bax, Bcl-2) were measured by RT-qPCR to explore the protective ability of AP-1-L on cells. After UVB irradiation, the relative expression level of proapoptotic factor in the model group were significantly increased, and the relative expression level of proapoptotic factor significantly decreased after AP-1-L irradiation. Taken together, AP-1-L is protective against cellular damage.

The Nrf2/Keap1 signaling pathway is involved in the body's antioxidant stress response. Nrf2 is one of the factors related to the oxidative stress response. Under normal conditions, Keap1 combines with Nrf2 to form a dimer, and the activity of Nrf2 is inhibited. When the body is damaged, the dimer dissociates, and Nrf2 enters the nucleus and binds with antioxidant elements to initiate downstream HO-1 expression, thereby maintaining the oxidation balance of the body and protecting the cells (Jin et al. 2014; Choi et al. 2014). In this study, the gene expression of Nrf2 was significantly down-regulated in HSF cells after exposure to UVA radiation, while the gene expression of Keap1 was significantly up-regulated and that of HO-1 was down-regulated. This suggests that UVA radiation promotes the dissociation of Nrf2 and Keap dimers. When AP-1-L was added and then UVA radiation was applied, gene expression of Nrf2 and HO-1 was up-regulated, while gene expression of Keap was significantly up-regulated, suggesting that AP-1-L enhanced activity of Nrf2 and downstream expression of HO-1 to maintain oxidative stress balance. The MAPK signal transduction pathway is involved in many cellular reactions. MAPK is able to transmit external stimuli to the nucleus through a transcriptional mechanism and is a key signaling molecule for UVA radiation (Gao et al. 2018). JNK, ERK, and p38 are three classic MAPK pathways (Nour et al. 2018). When exposed to UVA radiation, the MAPK pathway is activated by the phosphorylation of the ERK, JNK, and p38 proteins, and gene expression is up-regulated. c-Fos and c-Jun activities are activated upon phosphorylation of the MAPK pathway, further upregulating MAPK transcription (Zhao et al. 2023). In this study, the model group underwent UVA radiation, the MAPK pathway was phosphorylated, and the mRNA expression levels of related genes were elevated compared with the blank group, in which the overexpression of c-Fos and c-Jun induced the transcription of MMPs, which led to cellular damage and photoaging. When AP-1-L was added, the mRNA expression of related genes was down-regulated compared with the model group and even lower than that of the blank and positive control groups, suggesting that AP-1-L has anti-photoaging potential. In summary, it can be concluded that AP-1-L exerts antioxidant and anti-inflammatory effects by regulating the altered levels of genes related to the Nrf2/ Keap1/ HO-1 signaling pathway and the MAPK signaling pathway and has potential anti-photo-aging potential.

Based on the above discussion and experimental analysis, the anti-aging and anti-inflammatory effects of AP-1-L may be related to some indicators of aging and inflammation-related substances in skin cells, as well as the Nrf2/Keap1 signaling pathway and the MAPK signaling pathway. This study shows that AP-1-L at specific concentrations can not only promote cell proliferation but also protect and repair cells damaged by UV radiation. AP-1-L can reduce the ROS level in vivo, increase CAT activity and HA content, weaken MMPs expression, increase TIMP level, and increase COL-I content while reducing IL-6, IL-1β and TNF-α content in HaCaT cells. The apoptosis of HaCaT cells was inhibited by increasing the expression of the anti-apoptotic protein Bcl-2, inhibiting the expression of the proapoptotic protein Bax, and regulating the signaling pathways. By alleviating oxidative stress and reducing inflammation in epidermal cells, UV radiation damage to HSF cells and HaCaT cells can be prevented. AP-1-L has considerable application value. This study can provide valuable references for the further development of AP-1-L and new ideas for the R&D of natural anti-UV radiation substances.

## Conclusions

In summary, the experimental results of this study showed that AP-1-L had a protective effect on UV-damaged HSF and HaCaT cells, showing good anti-photoaging and anti-inflammatory effects. This effect may be achieved by regulating and improving some indicators of aging and inflammation-related substances in skin cells and the expression of Nrf2/Keap1 signaling pathway and MAPK signaling pathway-related genes. It has research potential in anti-photoaging and anti-aging, and then its application value can be determined by analyzing the specific composition of the lysate.

## Data Availability

The datasets used during the current study are available from the corresponding author on reasonable request.

## Abbreviations

AP-1-L: *Aureobasidium pullulans* Lysate
HSF: Human skin fibroblasts
HaCaT: Human immortalized keratinocytes
ROS: Reactive oxygen species
FBS: Fetal bovine serum
DMEM: Dulbecco’s modified Eagle’s medium
PBS: Phosphate buffered saline
CCK8: Cell Counting Kit-8
CAT: Catalase
HA: Hyaluronic acid
MMPs: Matrix metalloproteinases
MMP-1: Matrix metalloproteinase-1
MMP-2: Matrix metalloproteinase-2
MMP-3: Matrix metalloproteinase-3
MMP-9: Matrix metalloproteinase-9
TIMP: Tissue inhibitor of metalloproteinase
TNF-α: Tumor necrosis factor-α
IL- 6: Interleukin-6
IL-1β: Interleukin-1beta
Caspase-3: Cysteine aspartic acid specific protease-3
Caspase-9: Cysteine aspartic acid specific protease-9
BCL-2: B cell lymphoma factor-2
BAX: BCL-2-related X protein
COL-I: Human collagen-I
COL-III: Human collagen-III
ELISA: Enzyme-linked immunosorbent
RT-qPCR: Real-time quantitative PCR
